# Final height outcome of boys with idiopathic central precocious puberty treated with gonadotropin-releasing hormone analogue

**DOI:** 10.1186/1687-9856-2013-S1-P75

**Published:** 2013-10-03

**Authors:** Hua-mei Ma, Zhe Su, Qiu-li Chen, Yan-hong Li, Hong-shan Chen, Min-lian Du

**Affiliations:** 1 The first Affiliated Hospital of Sun Yat-Sen University, Guangzhou, China

## Aim

To observe the final adult height of 20 boys with idiopathic central precocious puberty (ICPP) treated with slow-releasing gonadotropin-releasing hormone analogue(GnRHa).

## Methods

Twenty boys with ICPP were treated with GnRHa for (20. 0 ± 6. 1) months. At the beginning of therapy, mean chronological age and bone age was(11. 4 ± 1. 0) years and(13. 0 ± 0. 4) years, respectively. GnRHa was discontinued when the boys reached the chronological age and bone age of (13. 2 ± 1. 1) years and (13. 7 ± 0. 6) years, respectively. At the conclusion of the study, all the boys had been followed up for (3. 3 ± 1. 5) years and had achieved adult height.

Comparisons were made among their predicted adult height(PAH), final adult height(FAH),and target height(THt). The long term outcome of final adult height in boys with ICPP was investigated after GnRHa treatment.

## Results

All the boys reached target height range. Final height was similar to the target height[(169. 8 ± 5. 8) cm *vs* (167. 8 ± 4. 6)cm, P>0. 05]. The height gain, defined as the difference between predicted adult height at the start of treatment using the height SDS for bone age and actual adult height was(3. 62 ± 3. 57)cm with the residual growth capacity of(11. 82 ± 3. 99)cm. PAH significantly improved after GnRHa treatment compared with before treatment [(169. 0 ± 5. 0)cm *vs* (166. 2 ± 4. 2)cm, P<0. 01]. There were no differences among PAH, FAH, and THt.

## Conclusion

GnRHa treatment can improve final height within the range of target height in boys with central precocious puberty.

